# Prevalence of the metabolic syndrome in patients with carotid disease according to NHLBI/AHA and IDF criteria: a cross-sectional study

**DOI:** 10.1186/1471-2261-12-2

**Published:** 2012-01-31

**Authors:** Milos Z Maksimovic, Hristina D Vlajinac, Djordje J Radak, Jelena M Marinkovic, Jagoda B Jorga

**Affiliations:** 1 Institute of Hygiene and Medical Ecology, Faculty of Medicine, University of Belgrade, Serbia; 2 Institute of Epidemiology, Faculty of Medicine, University of Belgrade, Serbia; 3 Department of Vascular Surgery, Dedinje Cardiovascular Institute, Faculty of Medicine, Belgrade, Serbia; 4 Institute of Medical Statistics and Informatics, Faculty of Medicine, Belgrade, Serbia

**Keywords:** metabolic syndrome, carotid disease, risk factors

## Abstract

**Background:**

Metabolic syndrome (MetS) has been related to type 2 diabetes and cardiovascular diseases. Different criteria for diagnosis of MetS have been recommended, but there is no agreement about which criteria are best to use. The aim of the present study was to investigate agreement between the National Heart, Lung, and Blood Institute, American Heart Association (NHLBI/AHA) and the International Diabetes Federation (IDF) definitions of MetS in patients with symptomatic carotid disease and to compare the frequency of cardiovascular risk factor in patients with MetS diagnosed by these two sets of criteria.

**Methods:**

The study was a cross-sectional one involving 644 consecutive patients with verified carotid disease who referred to the Vascular Surgery Clinic Dedinje in Belgrade during the period April 2006 - November 2007. Anthropometric parameters blood pressure, fasting plasma glucose and lipoproteins were measured using standard procedures.

**Results:**

MetS was present in 67.9% of participants, according to IDF criteria, and in 64.9% of participants, according to the NHLBI/AHA criteria. A total of 119 patients were categorized differently by the two definitions. Out of all participants 10.7% had MetS by IDF criteria only and 7.8% of patients had MetS by NHLBI/AHA criteria only. The overall agreement of IDF and NHLBI/AHA criteria was 81.5% (Kappa 0.59, *p *< 0.001). In comparison with patients who met only IDF criteria, patients who met only NHLBI/AHA criteria had significantly more frequently cardiovascular risk factors with the exception of obesity which was significantly more frequent in patients with MetS diagnosed by IDF criteria.

**Conclusion:**

The MetS prevalence in patients with symptomatic carotid disease was high regardless of criteria used for its diagnosis. Since some patients with known cardiovascular risk factors were lost by the use of IDF criteria it seems that NHLBI/AHA definition is more suitable for diagnosis of MetS. Large follow-up studies are needed to test prognostic value of these definitions.

## Background

During the last two decades metabolic syndrome (MetS) has become one of the major public-health challenges worldwide [1]. In 1988 Reaven was first to describe Syndrome X, and defined it as a cluster of hypertension, glucose intolerance, elevated tryglycerides and low level of high density lipoprotein (HDL) cholesterol [2]. Ferrannini et al. suggested that this clustering was caused by insulin resistance and called it insulin resistance syndrome [3]. World Health Organization (WHO) defined the syndrome and changed its name to metabolic syndrome [4]. In 2001 the National Cholesterol Education Program - Adult treatment Panel III (NCEP-ATP III) proposed both diagnostic criteria for metabolic syndrome and cut-off points for its components (abdominal obesity, hypertension, increased level of triglycerides and fasting plasma glucose and low level of HDL- C) [5]. NCEP-ATP III criteria were revised in 2005 by the American Heart Association (AHA) and the National Heart, Lung, and Blood Institute (NHLBI) - modified NCEP-ATP III criteria, called also NHLBI- AHA criteria [6], and in 2006 International Diabetes Federation recommended a new definition of metabolic syndrome - IDF definition [7].

According to the literature data, the prevalence of MetS depends on the definition used [8-11]. The concordance between various definitions has most frequently been high [9,10], although there are some subjects in whom MetS was diagnosed by one definition only and not by other(s). There is no agreement about which criteria for MetS diagnose are best to use.

The aim of the present study was to investigate agreement between the NHLBI/AHA and the IDF definitions of MetS in patients with symptomatic carotid disease and to compare the frequency of cardiovascular risk factor in patients with MetS diagnosed by these two sets of criteria.

## Methods

The study used for the present investigation was cross-sectional. It involved consecutive patients with verified carotid disease who referred to the Vascular Surgery Clinic Dedinje in Belgrade during the period April 2006 - November 2007. In the study were included 644 subjects who had symptoms of cerebral ischemia and carotid stenosis of ≥ 50%, according to NASCET criteria [12]. Carotid atherosclerosis was estimated by high resolution B-mode ultrasonography.

Patients under eighteen years of age, with malignant disease, previous endarectomy or rheumatoid arthritis, were excluded.

For all participants anthropometric parameters and data on cardiovascular risk factors were collected.

Waist circumference was measured at the midway between lower rib and crista iliaca according to WHO recommendations [13].

Body weight was assessed by using a calibrated standard balance-beam, height was measured by a standard height bar, and Body Mass Index (BMI) was calculated as weight (kg) divided by height (m^2^) [13]. Percent of body fat was assessed according to the method proposed by Durnin and Womerslaj [14].

Blood pressure was measured using the method recommended by the Seventh Report of the Joint National Committee on Prevention, Detection, Evaluation, and Treatment of High Blood Pressure [15].

For estimation of metabolic parameters, fasting plasma glucose (FPG) and lipoproteins, blood samples were obtained after an overnight fast and avoidance of liquids. Levels of fasting plasma glucose (FPG), total cholesterol (TC), serum triglycerides (TG), high-density lipoprotein cholesterol (HDL-C), low-density lipoprotein cholesterol (LDL-C) and serum uric acid level were estimated using commercial kits (Abbot, IL, USA) on an automated analyzer (AEROSET™, Abbot, IL, USA). Levels of high sensitivity C-reactive protein (hsCRP) and fibrinogen were measured by using Immunoturbidimetric fixed time test (Olympus Diagnostics, O'Callaghan's Mills Co. Clare, Ireland).

According to NHLBI/AHA criteria [6] metabolic syndrome was diagnosed when three or more of the following risk factors were present: abdominal obesity (> 102 cm in men, and > 88 cm in women), hypertension ≥ 130/≥ 85 mmHg or specific medication, level of triglycerides ≥ 150 mg/dl (1.7 mmol/L) or specific medication, low HDL cholesterol: in men < 40 mg/dl (1.03 mmol/L), and in women < 50 mg/dl (1.29 mmol/L) or specific medication, and fasting plasma glucose ≥ 100 mg/dl (5.6 mmol/L) or history of diabetes mellitus or taking antidiabetic medications.

According to the IDF criteria MetS include abdominal obesity (> 94 cm in men, and > 80 cm in women), plus any two of the same risk factors as in NHLBI/AHA criteria: hypertension ≥ 130/≥ 85 mmHg or antihypertensive therapy, level of triglycerides ≥ 150 mg/dl (1.7 mmol/L) or specific medication, low HDL cholesterol: in men < 40 mg/dl (1.03 mmol/L), and in women < 50 mg/dl (1.29 mmol/L) or specific medication, and fasting plasma glucose ≥ 100 mg/dl (5.6 mmol/L) or history of diabetes mellitus or taking antidiabetic medications.

## Statistical analysis

Categorical variables were presented by counts and percentages. Continuous variables were described as means with 95% confidence interval. Kappa statistics was used to measure agreement between the two criteria. ANOVA was applied for mutual comparisons of cardiovascular risk factors in subjects with MetS according to IDF, NHLBI/AHA and both criteria and subjects without MetS, as well as for comparison of cardiovascular risk factors among gender. Data were analyzed using SPSS package version 15 with significance level set to 0.05.

The study was reviewed and given ethical approval by the Ethics Committee at the Faculty of Medicine in Belgrade. All patients gave written, informed consent.

## Results

The study included 644 patients with symptomatic carotid disease, 402 (62.4%) males and 242 (37.6%) females (Table 1).

**Table 1:** Frequency of metabolic syndrome by IDF and NHLBI/AHA criteria

	Without MetS	MetS by both criteria	MetS by IDF only	MetS by NHLBI/AHA only	Chi square (*p *value) ^a^
	Number (%)	Number (%)	Number (%)	Number (%)	
All participants (n = 644)	157 (24.4)	368 (57.1)	69 (10.7)	50 (7.8)	
Gender:					
Male (n = 402)	113 (28.1)	203 (50.5)	42 (10.4)	44 (10.9)	
Female (n = 242)	44 (18.2)	165 (68.2)	27 (11.2)	6 (2.5)	28.39 (< 0.001)
Age group:					
35-54 (n = 78)	20 (25.6)	34 (43.6)	13 (16.7)	11 (14.1)	
55-64 (n = 199)	42 (21.1)	123 (61.8)	23 (11.6)	11 (5.5)	
65-69 (n = 144)	34 (23.6)	93 (64.6)	11 (7.6)	6 (4.2)	
70-74 (n = 142)	32 (22.5)	82 (57.7)	12 (8.5)	16 (11.3)	
≥ 75 (n = 81)	29 (35.8)	36 (44.4)	10 (12.3)	6 (7.4)	27.29 (0.007)

^a ^Chi-square tests was performed to assess variations in the distribution of patients among the four metabolic syndrome categories by sex and age.

Of 644 patients, 437 (67.9%) had metabolic syndrome according to IDF criteria and 418 (64.9%) according to the NHLBI/AHA criteria. The prevalence of metabolic syndrome was 60.9% in males and 79.3% in females according to the IDF criteria, and 61.4% in males and 70.7% in females according to the NHLBI/AHA criteria. A total of 119 patients were categorized differently by the two definitions. Out of all participants 69 (10.7%) had MetS only by IDF criteria (42 males and 27 females), and 50 (7.8%) patients had MetS only by NHLBI/AHA criteria.

The overall agreement of IDF and NHLBI/AHA criteria was 81.5% (Kappa 0.59, *p *< 0.001). There were significant differences in agreement between the two metabolic syndrome definitions based on sex and age. In males agreement was 78.6% (Kappa 0.55, *p *< 0.001) and in females 86.4% (Kappa 0.64 *p *< 0.001). For age groups agreement ranged from 69.2% (Kappa = 0.36, p < 0.001), for subjects 35-54 years old, to 88.2% (Kappa = 0.72, p < 0.001), for subjects 65-69 years old."

Comparisons of cardiovascular risk factors in patients with metabolic syndrome diagnosed by IDF and NHLBI/AHA criteria, and in patients without metabolic syndrome, are presented in Table 2 and Table 3.

**Table 2:** Mean values of cardiovascular risk factors in patients with metabolic syndrome according to IDF and NHLBI/AHA criteria and in patients without metabolic syndrome

	Without MetS	MetS by both criteria	MetS by IDF only	MetS by NHLBI/AHA only
	(n = 157)	(n = 368)	(n = 69)	(n = 50)
	Mean (95%CI)	Mean (95%CI)	Mean (95%CI)	Mean (95%CI)
Age (years)	65.7 (64.4, 67.1)	65.3 (64.5, 66.0)	63.7 (61.5, 65.9)	64.9 (62.5, 67.4)
Body mass index (kg/m^2^)	24.4 (23.9, 25.0)	29.0 (26.8, 27.4)	25.8 (25.3, 26.3)	23.4 (22.8, 24.1)
Waist circumference (cm)	89.9 (88.2, 91.7)	102.6 (101.7, 103.6)	92.0 (90.3, 93.6)	87.2 (85.6, 88.9)
% of body fat	27.7 (26.6, 28.7)	34.2 (33.6, 34.9)	30.8 (29.3, 32.2)	26.8 (25.4, 28.2)
Systolic blood pressure (mmHg)	138.1 (134.8, 141.2)	144.3 (142.4, 146.2)	141.9 (137.1, 146.7)	147.1 (141.2, 153.0)
Diastolic blood pressure (mmHg)	80.4 (78.6, 82.1)	84.1 (83.0, 85.0)	82.0 (79.2, 84.9)	81.7 (79.0, 84.3)
Triglycerides (mmol/L)	1.3 (1.2, 1.3)	2.2 (2.1, 2.3)	1.5 (1.4, 1.6)	1.9 (1.7, 2.1)
Total cholesterol (mmol/L)	5.2 (5.0, 5.4)	5.3 (5.2, 5.4)	5.1 (4.8, 5.3)	5.3 (4.9, 5.6)
HDL - C (mmol/L)	1.2 (1.1, 1.3)	1.0 (1.0, 1.0)	1.1 (1.0, 1.6)	0.9 (0.9, 1.0)
LDL - C (mmol/L)	3.5 (3.3, 3.6)	3.4 (3.3, 3.5)	3.2 (3.0, 3.5)	3.4 (3.1, 3.8)
Fasting plasma glucose (mmol/L)	4.8 (4.6, 4.9)	5.8 (5.6, 6.0)	5.0 (4.8, 5.2)	6.1 (5.4, 6.7)
hsCRP (mg/L)	4.7 (3.5, 5.9)	4.0 (3.4, 4.6)	3.9 (2.6, 5.3)	2.6 (1.7, 3.6)
Fibrinogen (g/L)	3.4 (3.3, 3.6)	3.4 (3.3, 3.5)	3.4 (3.1, 3.7)	3.2 (2.9, 3.5)
Serum uric acid (μmol/L)	315.2 (302.8, 327.6)	356.3 (346.6, 366.1)	325.0 (307.1, 342.9)	326.9 (302.0, 351.9)

HDL - C: High density lipoprotein cholesterol; LDL - C: Low density lipoprotein cholesterol; hsCRP: high sensitivity C reactive protein.

**Table 3:** Significant^a ^differences in cardiovascular risk factors in subjects with metabolic syndrome according to IDF, NHLBI/AHA criteria, and patients without metabolic syndrome

Variable			Metabolic syndrome according to:			
	Both criteria compared to:			IDF criteria only compared to:		NHLBI/AHA criteria only compared to:
	Without MetS	MetS by IDF only	MetS by NHLBI/AHA only	Without MetS	MetS by NHLBI/AHA only	Without MetS
Body mass index (kg/m^2^)	< 0.001	< 0.001	< 0.001	0.043	0.001	-
Waist circumference (cm)	< 0.001	< 0.001	< 0.001	-	0.034	-
% of body fat	< 0.001	< 0.001	< 0.001	0.003	0.004	-
Systolic blood pressure (mmHg)	0.006	-	-	-	-	0.003
Diastolic blood pressure (mmHg)	0.001	-	-	-	-	-
Triglycerides (mmol/L)	< 0.001	< 0.001	-	-	-	0.001
HDL-C (mmol/L)	< 0.001	0.037	-	0.029	0.015	< 0.001
Fasting plasma glucose (mmol/L)	< 0.001	0.001	-	-	0.002	< 0.001
Serum uric acid (μmol/L)	< 0.001	0.045	-	-	-	-

^a ^*p *value according to ANOVA; HDL - C: High density lipoprotein cholesterol

According to data presented in Tables 2 and 3, the greatest differences in cardiovascular risk factors were found between patients with MetS diagnosed by both criteria and patients without MetS. Patients with MetS were significantly more obese (BMI, WC and % of body fat) and had significantly higher mean values of sistolic and diastolic blood pressure, triglycerides, fasting blood glucose and serum uric acid and significantly lower mean value of HDL - C. In comparison with patients without MetS, patients in whom MetS was diagnosed only by IDF criteria had significantly higher mean values of BMI and % of body fat and lower values of HDL-C. Comparing with patients without MetS, those in whom MetS was diagnosed only by NHLBI/AHA criteria had significantly higher mean values of systolic blood pressure, triglycerides and fasting plasma glucose, and lower values of HDL-C. Significant differences in cardiovascular risk factors were found between patients with MetS diagnosed by only one of the two criteria and patients with MetS diagnosed by both criteria. In comparison with patients in whom MetS was diagnosed by both criteria, patients who met only IDF criteria were significantly less obese (lower mean values of BMI, WC and % body fat), had significantly lower mean levels of triglycerides, glucose and serum uric acid and higher mean level of HDL - C, while subjects who met only NHLBI/AHA criteria were only significantly less obese (lower mean levels of BMI, WC and % of body fat). In comparison with patients who met only NHLBI/AHA criteria, patients who met only criteria for IDF definition were significantly more obese (higher values of BMI, WC and % of body fat), but they had significantly lower mean level of glucose and higher mean level of HDL - C.

Patients who met only NHLBI/AHA criteria had significantly more frequently hypertension, elevated triglyceride and glucose level and a low HDL-C level in comparison with patients who met only IDF criteria (Table 4).

**Table 4:** Frequency of metabolic syndrome components in patients with metabolic syndrome according to IDF and NHLBI/AHA criteria

Metabolic syndrome component	Without MetS	MetS by both criteria	MetS by IDF only	MetS by NHLBI/ AHA only	*p *value^a^
	(n = 157)	(n = 368)	(n = 69)	(n = 50)	
	Number (%)	Number (%)	Number (%)	Number (%)	
Waist circumference ^b^:					
IDF	62 (39.5)	368 (100.0)	69 (100.0)	0 (0.0)	
NHLBI/AHA	35 (22.3)	297 (80.7)	0 (0.0)	0 (0.0)	
Triglycerides ^c^	10 (6.4)	248 (67.4)	17 (24.6)	30 (60.0)	< 0.001
Fasting plasma glucose ^d^	19 (12.1)	209 (56.8)	9 (13.0)	35 (70.0)	< 0.001
Hypertension ^e^	110 (70.1)	351 (95.4)	60 (86.9)	49 (98.0)	0.032
HDL - C ^f^	60 (38.2)	320 (49.7)	52 (75.4)	49 (98.0)	0.001

^a ^For comparison between IDF criteria only and NHLBI/AHA criteria only; *p *values were not calculated for waist circumference because of the basic differences between the two metabolic syndrome definitions.

^b ^> 94 cm in men and > 80 cm in women, by IDF criteria; > 102 cm in men and > 88 cm in women, by NHLBI/AHA criteria.

^c ^≥ 1.7 mmol/L or specific medication

^d ^≥ 5.6 mmol/L or history of diabetes mellitus or taking antidiabetic medication.

^e ^Systolic blood pressure ≥ 130 mmHg or diastolic blood pressure ≥ 85 mmHg or antihypertensive therapy.

^f ^High density lipoprotein cholesterol < 1.03 mmol/L in men, and < 1.29 mmol/L in women or specific medication.

Analyses of drug treatment in patients (antihypertensive, hipolipidemic and for diabetes mellitus type 2) did not change differences in observed cardiovascular risk factors between compared groups (data not shown).

## Discussion

In the present study metabolic syndrome prevalence in patients with symptomatic carotid disease was high regardless of criteria used for its diagnosis. According to IDF criteria MetS had 67.9% of patients, and according to the NHLBI/AHA criteria 64.9% of patients. The two definitions were concordant in 81.5% of patients (Kappa 0.59, *p *< 0.001). In comparison with patients who met only IDF criteria, patients who met only NHLBI/AHA criteria had significantly more frequently cardiovascular risk factors with the exception of obesity which was significantly more frequent in patients with MetS diagnosed by IDF criteria.

The high prevalence of MetS in this study could be expected since it is known that the MetS prevalence is high in population with verified atherosclerotic disease. For example, according to Gorter et al. study [16], the prevalence of MetS in various types of atherosclerotic disease was in range from 41% in patients with coronary heart disease, up to 58% in patients with peripheral arterial disease.

The high agreement of two definitions is also not surprising taking into account the fact that these definitions had the same five components and that four of these components are defined identically. IDF and NHLBI/AHA, vary in two important aspects. First, in the IDF criteria cut-off values for waist circumfernece are lower than in modified NCEP criteria (NHLBI/AHA), and the second and crucial, abdominal obesity is required as a prerequisite for diagnosis of MetS.

Similar results were found in other studies. The majority of these studies were performed in the samples of total populations and only few of them in patients with clinical atherosclerotic disease. In Li et al. study [10] the prevalence of MetS by NHLBI/AHA definition was 8.3% and by IDF definition 7.8%. The agreement was 94.4% in men and 97.0% in women. According to Athryos et al. [9] cross-sectional analysis of representative sample of Greek adults, the prevalence of MetS was 22.6% by NHLBI/AHA and 18.3% by IDF criteria. In a cross-sectional study among Malays in Kuala Lumpur, the MetS was diagnosed in 41.4% and 38.2% participants using the NHLBI/AHA and the IDF criteria respectively [11]. The use of the IDF MetS definition in the population of the United States [17], led to a higher prevalence estimate of the MetS than the use of NHLBI/AHA definition, although the two definitions similarly classified ~93% of the participants as having or not having the MetS. Out of four MetS definition (WHO, EGIR, NHLBI/AHA and IDF), applied in a population-based cross- sectional study in four population of the Asia-Pacific region [18], the highest prevalence of MetS was obtained with the IDF definition. Hildrum et al. [8] found that the prevalence of IDF-defined MetS was 29.6%, compared to 25.9% when using the NHLBI/AHA criteria. In a case-control study in which the association of first-ever acute ischemic non-embolic stroke with the MetS was investigated, the prevalence of MetS in the patients group was 57.1% by NHLBI/AHA and 69.9% by IDF criteria and 18.1% and 30.7% in the control group respectively. Among women with coronary artery disease, the prevalence of MetS was 70% and 74% by NHLBI/AHA and IDF criteria [19]. In all these studies differences in the prevalence of the MetS by two definitions were present, but they were not significant and not in the same direction. Yet, in some smaller or greater number of subjects, the MetS was diagnosed by only one of the two definitions.

What seems to be more important than the percentage of MetS diagnosed by one or the other definition, is which one of the two definitions better identifies subjects at high risk of cardiovascular diseases. According to Athryos et al. study [9], cardiovascular disease (CVD) prevalence was increased in the presence of MetS irrespective of the definition used, but this increase was more pronounced when NHLBI/AHA criteria were applied in comparison with IDF definition. NHLBI/AHA criteria were also better, in comparison with IDF criteria, in identifying the metabolically abnormal, but non-obese, groups known to be predisposed to type 2 diabetes and cardiovascular diseases [20]. Li et al. [10] found that in the patients with hypertension, the IDF defined MetS was more strongly associated with coronary heart disease than NHLBI/AHA defined MetS, but it was weakly or not associated with stroke. Milionis et al. [21] reported that the implementation of the IDF MetS definition, although substantially increased the number of elderly subjects labeled as having MetS, did not contribute to the identification of those at high risk of stroke. Comparing prognostic utility of the two definitions of MetS in postmenopausal women with angiographic coronary heart disease, Brown et al. [19] stated that their analysis suggested that both MetS definitions successfully identified a subset of women at high risk for future cardiovascular events. However, the number of patients differentially classified by the two definitions was small and there were few cardiovascular events overall, so that they were unable to draw firm conclusions about differences in the clinical characteristics or risk for cardiovascular clinical outcomes between these two definitions.

In the present study we did not follow up the participants so we cannot be sure about prognostic value of any of the two definitions. However, cardiovascular risk factors were more frequently present or were more adverse in patients with MetS who met only NHLBI/AHA criteria than in those who met only IDF criteria. Patients who met only NHLBI/AHA criteria differ less from patients whose MetS was diagnosed by both definitions than patients who met only IDF criteria. Since some patients with known cardiovascular risk factors were lost by the use of IDF definition it seems that NHLBI/AHA definition is more suitable for diagnosis of MetS. Similar to our findings, in the cross-sectional study in Malay cohort, Moy and Bulgiba [11] found that blood pressure, glucose, total cholesterol and triglycerides were more adverse in participants diagnosed by NHLBI/AHA criteria than in the IDF group. They concluded that their results suggested that central obesity should not be the prerequisite for the development of increased cardiometabolic risks and that for diagnosis of MetS was better to use NHLBI/AHA than IDF criteria, at least in their own population.

The main limitation of this study is its cross-sectional design. Prospective studies are needed to find out which of the two definitions, IDF or NHLBI/AHA, better determines adverse events associated with the MetS.

## Conclusion

The MetS prevalence in patients with symptomatic carotid disease was high regardless of criteria used for its diagnosis. The two definitions were concordant in 81.5% of patients, but since some patients with known cardiovascular risk factors were lost by the use of IDF definition, it seems that NHLBI/AHA definition is more suitable for diagnosis of MetS. Large follow-up studies are needed to test prognostic value of these definitions.

## Competing interests

The authors declare that they have no competing interests.

## Authors' contributions

MM and HV participated in the design of the study and in analysis and interpretation of data, and drafted the manuscript. DR and JJ participated in the design of the study. JM performed statistical analysis and participated in interpretation of data and drafting of the manuscript. All authors read and approved the final manuscript.

## Pre-publication history

The pre-publication history for this paper can be accessed here:

http://www.biomedcentral.com/1471-2261/12/2/prepub
